# Dyadic Associations of Depressive Symptoms and Cardiovascular Health in Middle-Aged and Older Couples

**DOI:** 10.1093/geroni/igaf122.237

**Published:** 2025-12-31

**Authors:** Yenee Soh, Richard Jones, Izzuddin Aris, Ichiro Kawachi, Henning Tiemeier

**Affiliations:** Harvard T.H. Chan School of Public Health, Boston, Massachusetts, United States; Brown University, Providence, Rhode Island, United States; Harvard Medical School, Boston, Massachusetts, United States; Harvard T.H. Chan School of Public Health, Boston, Massachusetts, United States; Harvard T.H. Chan School of Public Health, Boston, Massachusetts, United States

## Abstract

Depressive symptoms contribute to worse cardiovascular health, yet the role of spousal depressive symptoms on this association remains unknown. We examined the longitudinal association between depressive symptoms and cardiovascular health (CVH) in spousal dyads within the Health and Retirement Study (HRS), a cohort of U.S. adults aged 50+ years. Among 4,360 heterosexual spousal dyads, depressive symptoms were assessed at baseline (Wave 1) using the modified 8-item Center for Epidemiologic Studies-Depression scale. The HRS collected sociodemographic covariates at baseline (age, race and ethnicity, and education) and measures of body mass index, blood pressure, fasting glucose, total cholesterol, and smoking (Waves 2-4), which were used to calculate a CVH score (range 0-100 points; higher scores reflect better CVH) as defined by the American Heart Association. We used the Actor-Partner Interdependence Model and latent growth curve modeling to examine how both spouses’ depressive symptoms were associated with subsequent CVH, adjusting for covariates. For men, their own (B=-1.27; 95%CI:[-1.65,-0.89]) and their spouse’s depressive symptoms (B=-0.42; 95%CI:[-0.74,-0.11]) at baseline were associated with their own CVH at Wave 2. Similar associations were observed for women’s own and their spouse’s depressive symptoms on their CVH. Additionally, only husbands’ depressive symptoms were associated with a faster decline in wives’ CVH (B=-0.08; 95%CI:[-0.14,-0.03]) over an 8-year period (Waves 2-4). Our findings suggest both an individual’s and their spouses’ depressive symptoms may mutually influence long-term CVH. Addressing depressive symptoms within the spousal context may be a strategy to improve CVH and reduce cardiovascular disease risk in middle-aged and older couples.

